# ANA Webinars: Clinical and basic research resilience during COVID‐19

**DOI:** 10.1002/acn3.51230

**Published:** 2020-12-15

**Authors:** Michelle C. Johansen, Michelle Jones‐London, Walter Koroshetz, Eliezer Masliah, Avindra Nath, Vicky Whittemore, Justin C. McArthur

**Affiliations:** ^1^ Department of Neurology The Johns Hopkins University School of Medicine Baltimore Maryland USA; ^2^ The National Institute of Neurological Disorders and Stroke Bethesda Maryland USA; ^3^ Division of Neuroscience National Institute on Aging Bethesda Maryland USA

## Introduction

These are unprecedented times for academic researchers, with many struggling to make sense of the current pandemic and experiencing anxiety over how their careers may be impacted. As a result of COVID‐19, early‐career investigators may feel isolated or fearful, as their options for alternate funding or research can be limited when compared to an established researcher. In this live webinar (5‐8‐2020), produced by the ANA (https://vimeo.com/416480771), experts and leaders from the National Institute of Health (NIH) joined together to provide the most up‐to‐date and accurate information regarding how a junior investigator can stay mentally, emotionally and scientifically resilient in their research endeavors during COVID‐19. The following statements summarize the key points from the discussion.

1. **The National Institute of Neurologic Diseases and Stroke (NINDS) and the National Institute of Aging (NIA) are committed to accommodating research that is not COVID‐19 related.**

The support for early‐career investigators, and the recognition that this is a trying time for clinical and basic science researchers, was unanimous among all participants on the panel. Dr. Koroshetz (Director of NINDS) begins the discussion by encouraging the listener to remember the mission that originally brought physician‐scientists into the field of neuroscience: to alleviate the suffering of those with neurological disease and developmental disorders. The NINDS and NIA recognize that debilitating neurologic conditions continue for patients, despite the materialization of COVID‐19, and the overall goal of the institutions remains the same. Dr. Nath’s (Chief section of Neurological Infection, Clinical Director, NINDS) perspective is that the current priority of the intramural NINDS is similar to academic institutions, with the main concern being keeping patients and physicians safe, even while trying to conduct the most rigorous research.

1. **The difficulties in conducting clinical and bench research experienced by junior investigators during COVID‐19 are also experienced by senior investigators at the NIH.**

A reverberating message from the discussion is that early‐career investigators are not alone in their struggles. The senior investigators at both the intramural NINDS and NIA are having to develop novel techniques to continue non‐COVID‐related research. Dr. Masliah (Director of NIA) articulates that despite the increase in funding for Alzheimer’s related research, many research programs are currently at a standstill. He recognizes the gravity of the delay as the institution has committed to developing solutions to Alzheimer’s and related illnesses by 2025. He states that even the NIA was aware of the implications of not being able to obtain longitudinal follow‐up data or autopsy specimens, critical to aging research. Dr. Jones‐London (Chief of Programs to Enhance Neuroscience Workforce Diversity, NINDS) encourages investigators to reach out to the extramural departments and program officers during this time, stating that they are open, and committed to helping early‐career investigators. Dr. Koroshetz agrees, stating that the work on the extramural side has been moving along at its normal pace as a result of tele‐technology, and they are committed to reviewing grants and distributing funds as planned.

1. **There are ways to conduct non‐COVD‐related research during COVID‐19.**

Many on the panel encourage early‐career investigators to become creative, and to view some of the developments in technology as blessings in disguise for conducting research during COVID‐19. These rapid accelerations enable researchers to keep participants safe while continuing research outside of the hospital. Dr. McArthur (Chief of Neurology, Johns Hopkins, President of ANA) recommends using the telemedicine platforms, which have been developed for clinical care, for research purposes. Dr. Nath adds that the NINDS has been leveraging remote patient consent and obtained approval for this technique from their institution review board. Dr. McArthur outlines some strategies researchers could consider while reopening, such as shift work in the laboratory, putting cleaning materials at common workstations and using Plexiglas shields to help prevent transmission. Drs. Nath and McArthur recognize that the limitations faced by basic science researchers will differ from clinical researchers, but given the unknown duration of COVID‐19, developing innovative strategies early will lead to resilience, rather than anticipating that the research environment will return to normal rapidly. Dr. Masliah suggests a mixed approach to research, where interaction with participants is done safely, but other technologies are leveraged to follow longitudinal outcomes. He highlights the many data repositories that are available for researchers to conduct virtual experiments that may never have been accessed previously as a result of time constraints.

1. **The agencies are working to be as accommodating as possible with questions, new grant submissions and research deadlines.**

Dr. Jones‐London emphasizes that flexibility is the key word spoken at the NINDS and that talking to your program officer is always welcome. Dr. Whittemore (Program Director, NINDS) speaks from personal experience, citing examples of ways she has been providing support to individuals who are looking for answers, such as discussing ways to continue ongoing research projects during COVID‐19, or finding alternate funding sources. She encourages others, as Dr. Jones‐London does, to reach out to their program officer. Dr. Koroshetz reminds the group of the recent extension of deadlines and eligibility time windows for K awards and Early Investigator Status (5/8/2020, https://grants.nih.gov/faqs#/covid‐19.htm). He anticipates this continuing in the future, stating that the commitment of the NINDS is to “help people stay in the game and get back in the game.”

1. **The experts offer suggestions for how to overcome institution‐specific problems encountered by the early‐career investigator**

In response to several concerns expressed by participants about difficulties within their own institution, Dr. Whittemore states that based on talking to many individuals across the United States, most institutions are developing plans to ease back into full research endeavors. It appears that a phased return to work approach is the most successful. She anticipates that leadership will continue to distribute these plans to early‐career investigators. Dr. McArthur offers additional insight as a department chair, stating that junior investigators should not be afraid to ask leadership for a plan on how they anticipate returning to work, and even engage in the development of the plan.

1. **It is not necessary to pivot your research to COVID‐19‐related research**

The panel agrees that having an established, successful research career is not simply about chasing high priority funding areas. Dr. Jones‐London cautions that to build your career on something that the investigator is neither passionate about, or has the skill set to obtain, is not a good approach. There are also ways to consider ones current line of research in light of COVID‐19, rather than stopping ones project and pivoting completely to COVID‐19. Dr. Whittemore again encourages junior investigators to reach out to their program officers, even just to talk through different ideas, recognizing that while it may not be for the rest of the person’s academic career, there may be ways to take advantage of COVID‐specific funding opportunities for ongoing projects.

1. **However, it may be that COVID‐19‐related research is a critical opportunity and could change your career trajectory**

Dr. Koroshetz, Dr. McArthur and Dr. Nath all spoke about the impact that acquired immunodeficiency syndrome (AIDS) had on their careers, as both physicians and as clinician‐scientists, recalling that there was great hesitancy on the part of many neurologists to attempt AIDS‐related research. Dr. Koroshetz encourages junior investigators to consider all avenues of COVID‐related research, from the biological effects on the nervous system to the long‐term effects of the virus. Many point to the wealth of information that is available on the NIH website, for those who are interested in acquiring more information.

1. **Funding for a career development grant with a favorable score should not be limited by COVID‐19**

Dr. Koroshetz clarifies that there is no percentile by which K awards are funded, but reiterates that the NIH is continuing to evaluate for funding all grants considered as outstanding or excellent by study section. The NIH institutes are committed to early‐career investigators, whose R01 grants are funded up to 10 percentile points above payline for established scientists. Dr. Masliah states that the future is uncertain right now for everyone, so submit the grants now rather than waiting. He reiterates that the K funding line at the NIA currently is strong, and that the extramural division was working tirelessly to review all submitted grants. Dr. Whittemore adds that study sections are not allowing the pandemic to delay scoring of applications.

## Conclusion

At the end of the webinar, each expert is asked to offer one piece of advice to junior and early‐career investigators on how to stay “research resilient” during COVID‐19. Dr. Jones‐London states that there is a path forward, but do not be afraid to ask questions, as not asking will never result in answers. She encourages the audience to take advantage of online professional development tools now, while investigators have the time. Dr. Koroshetz encourages ingenuity, rather than waiting for things to change, using the downtime to plan more effectively, and learn important skills. Dr. Whittemore reiterates that program officers are available to talk through all ideas. Dr. Nath quotes Charles Darwin who said, “It is not the strongest of the species nor the most intelligent that survives. It is the one most adaptable to the changing environment in which it finds itself.” While everyone will face difficult situations that can appear hopeless, he states that it is possible to emerge stronger. Dr. Masliah reminds everyone that neuroscience is an area that is resilient and imaginative by its very nature, and that utilization of the new technology previously mentioned will lead to resilience. Finally, Dr. McArthur encourages everyone to step outside of their comfort zone, not remaining tied to their field of expertise, while continuing to build networks to strengthen their research.

## Conflicts of Interest

The authors reports no relevant conflicts of interest.

